# Dizziness and neck pain: a perspective on cervicogenic dizziness exploring pathophysiology, diagnostic challenges, and therapeutic implications

**DOI:** 10.3389/fneur.2025.1545241

**Published:** 2025-03-17

**Authors:** Willem De Hertogh, Alessandro Micarelli, Sue Reid, Eva-Maj Malmström, Luc Vereeck, Marco Alessandrini

**Affiliations:** ^1^Faculty of Medicine and Health Sciences, Research Group MOVANT, University of Antwerp, Antwerp, Belgium; ^2^Unit of Neuroscience, Rehabilitation and Sensory Organs, UNITER ONLUS, Rome, Italy; ^3^Faculty of Health, School of Allied Health, Australian Catholic University, North Sydney, NSW, Australia; ^4^Department of Otolaryngology, Clinical Sciences, University of Lund, Lund, Sweden; ^5^Department of Clinical Sciences and Translational Medicine, Ear-Nose-Throat Unit, University of Rome Tor Vergata, Rome, Italy

**Keywords:** dizziness, cervicogenic dizziness, neck pain, vertigo, cervical

## Abstract

Dizziness and vertigo affect up to 20% of adults annually. Cervicogenic dizziness (CGD), a debated clinical entity, is characterized by dizziness associated with cervical pain or dysfunction, stemming from altered proprioceptive input from the cervical spine. Despite its recognition in clinical practice, CGD remains controversial due to its reliance on exclusionary diagnosis and the absence of specific diagnostic criteria or gold-standard tests. This perspective explores the pathophysiology of CGD, emphasizing the sensory mismatch theory, where disrupted cervical proprioception interacts maladaptively with vestibular and visual systems, leading to postural instability and dizziness. Central mechanisms, including sensory reweighting and maladaptation, further complicate symptom resolution, particularly in the context of chronic cervical dysfunction. Current diagnostic approaches provide insights but lack specificity. Management strategies, including manual therapy and sensorimotor exercises, show promise in alleviating symptoms by targeting cervical dysfunction and enhancing proprioceptive integration. However, these interventions highlight the need for an integrated approach that addresses both cervical and vestibular contributions to dizziness. This paper underscores the importance of advancing CGD research, particularly understanding central maladaptation mechanisms. By bridging gaps in clinical and research knowledge, a more comprehensive framework for diagnosing and managing CGD can emerge, benefiting patients with persistent dizziness and cervical involvement.

## 1 Introduction

Dizziness and vertigo are common symptoms affecting about 15 to 20% of adults yearly in large population-based studies (1). The most common causes are peripheral vestibular conditions and cardiovascular diseases (2). Physiological causes are many and diagnosis involves a thorough patient history and clinical examination, which may include multiple testing from clinical examinations to neuro-otological evaluations and brain scans (3). Frequently, no single cause can be determined (4), and multiple diagnoses may overlap (5). Often, various factors converge, sometimes alongside comorbidities (6, 7).

Dizziness can become persistent due to diagnostic or therapeutic challenges or when symptoms do not resolve naturally (8). Persistent dizziness is difficult to treat, since there are multiple possible mechanisms that can lead to chronicity.

Identifying these mechanisms is essential for tailored treatment (9). Vestibular compensation is a crucial mechanism in the natural resolution of symptoms. A triggering event may initiate a compensation phase with transient behavioral and perceptual changes (10). However, compensatory mechanisms may become maladaptive. These maladaptive processes involve abnormal interactions between the visual, somatosensory, and vestibular cortices, as well as higher executive areas, limbic structures and motor efferent regions (11). Consequently, maladaptive sensory reweighting and mismatches between expected and actual motion signals, along with heightened introspection, can lead to symptoms such as visual induced dizziness, movement induced dizziness, unsteadiness, gait disorders, cognitive fatigue and avoidance behaviour (12–14).

Recently, criteria for diagnosing long-standing conditions, such as persistent postural-perceptual dizziness (PPPD), have been proposed, emphasizing the need to address multiple contributing factors such as anxiety-related personality traits, heightened anxiety and vigilance during precipitating events, alteration in postural control strategies, shifts in multisensory integration and reduced cortical integration of spatial orientation and threat assessment networks (15). Patients with dizziness often complain of neck pain and neck pain and dizziness frequently coincide with or without a causal relationship (16). For instance, 57% of patients referred to a vestibular unit for dizziness and balance disorders reported neck and shoulder pain (17). Knapstad et al., found that 43% of patients with long-lasting neck pain reported dizziness (18), which is a higher prevalence than the general population’s 29.3 to 32% (1). Similarly, in patients with dizziness, neck pain was present in 37.5% of those with BPPV and 46.7% with other dizziness causes (19), compared to the point prevalence of neck pain of 3.5 to 5% in a general population (20). In summary, muscular skeletal pain often coexists with dizziness (6).

In patients with dizziness and neck pain, vestibular migraine has been suggested as the most probable cause (21). Diagnoses like cervicogenic dizziness still remain controversial and are often not considered in vestibular settings, although projects are ongoing to develop structured consensus diagnostic criteria within the International Classification of Vestibular Disorders (ICVD) (21–24). Patients with dizziness can develop secondary neck pain, due to avoidance behaviour and head-on-trunk stiffness in order to reduce head movements, leading to the misperception that the cervical region is the cause of dizziness. Yet, neck pain can be a perpetuating factor in dizzy patients. It may reduce cervical range of motion, and neck stiffness, associated with the reduced head movements, has been linked to increased susceptibility to BPPV recurrence and failure of repositioning manoeuvres (19, 25, 26). Studies show that people with idiopathic neck pain have increased postural sway (27–30). Consequently, postural sway improves after manual therapy in patients with dizziness of suspected cervical origin (31). Overall, several physiological facts underscore considering a relationship between neck pain, cervical proprioception, and balance, well supported by theories on the interaction between cervical proprioception and the vestibular organs (32–34).

Our aim is consequently to reflect critically on the concept of cervicogenic dizziness as a form of persistent dizziness and to provide an overview on the pathophysiology with implications for its management and research.

## 2 Cervicogenic dizziness

### 2.1 Definition

Cervicogenic dizziness can be defined as a non-rotatory dizziness, associated with neck pain and/or reduced neck mobility. It can be provoked by cervical movements or positions, i.e., head movements relative the torso. Patients experience a feeling of light-headedness, giddiness, unsteadiness or a feeling of imbalance (35, 36). Prevalence numbers vary from 5 to 6% in Ear-Nose-Throat practices (37, 38) to 40% in patients with neck pain (39). Patients with cervicogenic dizziness show Dizziness Handicap Inventory scores comparable to those observed in other forms of chronic dizziness (40).

It can be approached within a broad definition, including vascular diseases as well as secondary vascular disease caused by cervical spondylosis, as well as sensory mismatch due to impaired cervical proprioception (41, 42). Most reports and studies rely on a narrower definition based on a sensory mismatch hypothesis caused by cervical proprioceptive impairment. In the current manuscript we adhere to this narrower definition. Indeed, with proprioceptive cervicogenic dizziness there are often altered impulses from cervical proprioceptors in the deep cervical muscles, joints, discs and ligaments (42).

The sensory mismatch theory builds on impaired peripheral afference (bottom-up inputs) and on central maladaptation (top-down signals). Visual, vestibular, somatosensory systems and cervical proprioception, needed to maintain balance, need to be perceived, integrated, and interpreted by the brain to generate appropriate motor responses (43, 44).

### 2.2 Pathophysiology

#### 2.2.1 Evidence from lab studies

The importance of cervical proprioception in balance control has been studied extensively.

In 1856 Claude Bernard described the role of proprioception, central nervous processing, and integration of vestibular and visual cues in sensorimotor control of the head and body, noting balance loss in dogs after deep cervical muscle transection (45). More recently, Sadeghi et al. (46) demonstrated that cervical proprioception can compensate for the loss of vestibular input after labyrinthectomy in alert monkeys, even at the single neuronal level of the vestibulo-ocular reflex arc. These monkeys, after contralateral labyrinthectomy, could perform head movements as precisely as healthy controls. Neurons in the vestibular cerebellum, processing either vestibular input or combined vestibular and proprioceptive input, can distinguish between active and passive head movements and differentiate between body movements under a stationary head and head movements on the body (47).

Local anaesthetic injections in the neck have caused nystagmus and ataxia in animals, and ataxia and a sense of tilting without nystagmus, in humans. Unilateral disconnection of C1–C3 dorsal roots nearly replicates the effects of a unilateral labyrinthectomy and unilateral transection of the upper cervical afferents, leading to severe ataxia and nystagmus (48–50).

Neck muscle vibrations in humans can cause prolonged eye position changes (51), visual illusory movements (52), and increased body sway (53, 54) and alterations in orientation and gait pattern (55, 56).

This illustrates that alterations in cervical proprioception affect balance, oculomotor control and orientation and perception of verticality.

#### 2.2.2 From cervical proprioceptive afference to pain-induced impairments

Cervical proprioceptive afference originates from muscle spindles and mechanoreceptors (57–60). Combined with vestibular organs’ ability to encode movements (61), cervical afference is crucial for processing vestibular data and maintaining balance (23, 62). Although it is clear from studies that the semicircular canals play a key role in disambiguating tilt and translation, many studies have suggested neurons encoding tilt are likely influenced by extra vestibular signals (63). Indeed, one possible mechanism is that the fore-aft translation was sensed by somatic sensation in the body rather than the labyrinth and that neck proprioception is used as an indicator of head tilt allowing acceleration sensed in the otoliths to be converted into body coordinates (64). The abundance of local muscle spindles in upper cervical segments highlights its importance. Dysfunctional joints may alter Type 1 cervical articular mechanoreceptors and proprioceptors, leading to a loss of normal afferent input (65). This aberrant information interacting with the vestibular nuclei (66), potentially might cause dizziness. Sensory mismatch between cervical proprioceptive afference and visual and vestibular sensations could be one cause of dizziness and imbalance in patients with cervical spine symptoms (67). However, the literature diverges on whether and how neck pain can alter cervical proprioception. Furthermore, most subjects with neck pain do not experience dizziness, therefore some other factors have to be contributing, e.g., sensitivity for sensory inputs as found in chronic pain patients (68).

Cervical proprioception is commonly assessed using cervical repositioning or relocation tasks, where individuals are asked to return their head to a predefined position after an active movement. The main outcome measure in these tasks is joint position error, or repositioning error, which quantifies the accuracy of the repositioning effort and reflects proprioceptive function (69).

In asymptomatic controls, studies with experimentally induced muscular fatigue and pain have shown both decreased and increased accuracy in sensorimotor control, suggesting cervical impairments can alter proprioception both ways (70, 71), and provocation of dizziness in some subjects (71).

One major concern is the likelihood that pain can alter cervical proprioception. Seminal studies have found that subjects with cervical pain exhibit decreased sensorimotor control (44, 72–75), especially in those with frequent pain (76). These findings support the notion that pain can alter cervical proprioception and cervical afference, fostering a sensory mismatch.

Clinical conditions such as neck trauma, neck muscle spasms, fatigue, cervical degenerative disease or chronic pain can alter proprioceptive inputs, leading to dizziness and instability (35, 77, 78).

Acute conditions like whiplash injuries can damage proprioceptive receptors in facet joints, discs, and muscles (41), and neck injuries can impair balance and vision due to impaired proprioception (42). Interstitial inflammatory mediators produced by muscle fatigue can sensitize muscle spindles (79). In case of inflammation, the overactivation of mechanoreceptors in the cervical intervertebral discs and facet joints control, along with a significant increase in their number, is believed to cause erroneous proprioceptive signals. These mechanoreceptors typically monitor the activity of muscle spindles and paraspinal muscles (80). However, when overactivated during inflammation, they may contribute to distorted proprioceptive feedback (81, 82).

Chronic conditions, such as neck pain, are associated with functional and morphological muscle changes, which can alter the firing rates of Golgi tendon organs and muscle spindles, affecting proprioception (62, 83). In cervical degenerative diseases associated with neck pain, muscle fatigue, stiffness or dizziness (84–86), a multitude of mechanoreceptors, including Ruffini corpuscles and nociceptive receptors, have been found growing into the degenerative cervical intervertebral disk, contributing to pain (81, 82, 87).

Patients with cervicogenic dizziness have been found to improperly perform the cervical relocation test (CRT, see paragraph below for details) (40) and the rod and disk test, linking pain and cervical proprioceptive deficits but also suggesting maladaptive sensory reweighting and indicating a derangement of cervical proprioception and increased visual dependence (88). This supports the theoretical model, linking pain and cervical proprioceptive deficits (73, 76).

#### 2.2.3 Central mechanisms, maladaptation, and sensory reweighting

**Figure 1:**
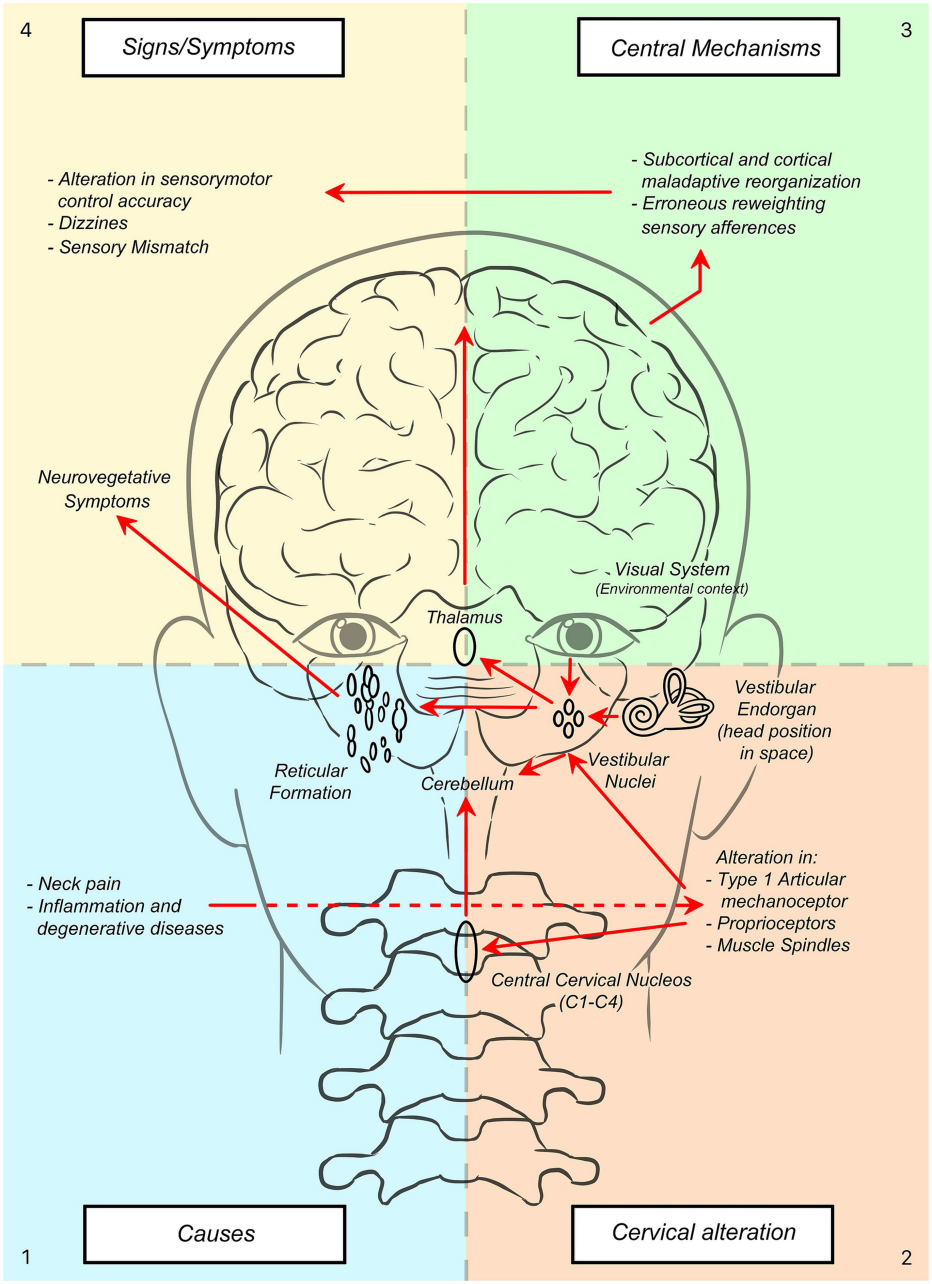
How causes (1) can lead to signs/symptoms (4) in cervicogenic dizziness. A schematic overview of pathophysiological and central adaptive mechanisms.

The brain’s ability to utilize multiple information sources, such as vestibular, visual, auditory and proprioceptive signals from the lower limb and neck, prevents disturbances in one system, such as the neck, from necessarily causing dizziness (61). Cervical proprioceptive signals converge with vestibular and visual inputs at the vestibular nuclei, thalamus, and cerebral cortex in the robust postural control system (34, 89–93), as presented in Figure 1 by Treleaven (44).

Animal studies highlight that cervical proprioceptive afferents project to the central cervical nucleus through the dorsal root ganglion which then convey to the cerebellum and reticular formation (92–94). This underscores the need for a broader understanding of the cervical spine’s role in postural control, as its proprioceptors interact with the vestibular and visual systems at multiple levels of the central nervous system to coordinate movements through various reflexes (86, 90).

Cervicogenic dizziness often includes autonomic symptoms, such as palpitations, nausea, and vomiting. This can be due to connections between the spinal cord to the vestibular nuclei via the cerebellum and reticular formation (95). The vestibular nuclei also send inputs to the reticular formation and parabrachial nucleus, which project to sympathetic preganglionic neurons in the thoracic spinal cord, adjusting circulation, digestion, and respiration for homeostasis through the vestibulo-sympathetic reflex pathway (96, 97). Sensory mismatch between vestibular, visual, and cervical proprioceptive systems affects the function of the reticular formation and parabrachial nucleus, leading to abnormal sympathetic outflow and associated cardiovascular and gastrointestinal symptoms. Sympathetic innervation is directly related to the intrafusal fibres (98), and sympathetic outflow intensely inhibits proprioceptive input cervical muscle spindles in cats (99).

The vestibular system informs head position in space, while the visual system provides environmental context (100), allowing for coordinated motor responses within a body-centred frame of reference (101). However, cervical pain can distort sensorimotor control, leading to long-term effects on proprioception and central reorganization (71, 102). This maladaptive reweighting of afferent input may contribute to the visual dependence observed in patients with neck pain and cervicogenic dizziness (40, 88, 103–105).

In summary, altered cervical proprioceptive inputs due to cervical impairments can result in sensory mismatch and central maladaptation, which can lead to cervicogenic dizziness or dizziness influenced by the cervical spine (see Figure 1).

## 3 Implications for the management of dizzy patients

### 3.1 Diagnosing cervicogenic dizziness

For the diagnosis of cervicogenic dizziness specific tests are non-existent. Despite many efforts to develop them, they are still absent due to the lack of a gold standard (106–108). As an alternative, clinical reasoning algorithms have been developed and suggested to handle the entity (22, 36). These algorithms consider cervicogenic dizziness to be a diagnosis of exclusion, meaning that first other causes of the dizziness such as dysfunction of the central nervous system, cardiovascular system, and the peripheral vestibular system need to be ruled out (35, 36, 67, 108). Therefore, in this manuscript we consider these vestibular and other causes to be excluded.

#### 3.1.1 History taking

A thorough patient history is crucial for the differential diagnosis of dizziness. Asking the right questions can guide the diagnosis, which should then be confirmed through additional physical testing. Several core sets of questions have been proposed to guide this history taking (11, 109, 110). When taking a history, specific symptoms related to common vestibular disorders should be explored.

Clinicians evaluating patients suspected of cervicogenic dizziness should focus on specific factors. They should inquire about symptoms such as feelings of imbalance or unsteadiness, as opposed to vertigo, which is characterized by a sensation of spinning or rotatory motion. The dizziness is usually provoked by cervical movements or positions. There must be a presence of neck symptoms such as pain or stiffness. A temporal relationship between dizziness and neck pain, in terms of start and duration of both, is considered an important factor, where neck pain shall precede dizziness. The intensity of the neck pain and dizziness are usually related. Symptom presentation is usually episodic or intermittent, lasting from minutes to hours, but may persist for days, months, or even years. Some patients may have a traumatic cause, while in others, the condition may be idiopathic (35, 36, 111).

The value of these questions to be diagnosis specific are questioned, as symptoms like light-headedness can also be associated with various vestibular disorders. Indeed, interpreting patient responses requires caution, as patients often struggle to accurately report their symptoms (112). Again, the lack of a gold standard for cervicogenic dizziness makes it hard to underpin the diagnostic value of items of the patient history. Despite these challenges, recognizing these symptoms remains important. The absence of typical vestibular symptoms decreases the likelihood of them being present. For example, when comparing 25 patients with CGD with 25 patients with BPPV, those with CGD reported more a feeling of light-headedness than those with BPPV (108).

In cases of cervicogenic dizziness with a traumatic onset, such as whiplash-associated disorders (WAD), dizziness is common in patients with chronic WAD. One might consider these patients as being concussed, and consequently dizzy. However, since vestibulopathy as assessed via caloric testing at that time was detected in only a minority of WAD cases, post-traumatic dizziness seems to be primarily related to the cervical spine rather than a concussion affecting the vestibular system (113). The contribution of the cervical spine is confirmed by Treleaven et al. who found that the lack of improvement on cervical spine parameters correlated with persistent dizziness after WAD (114). Importantly, dizziness after trauma can be multifactorial and should not be regarded as caused by one system (115). Post-traumatic BPPV should always be ruled out initially.

#### 3.1.2 Physical examination

In patients with suspected cervicogenic dizziness, a physical examination directed to the (upper) cervical spine is essential to identify potential cervical spine dysfunction. To assess how well the cervical spine integrates with other components of the balance system, this examination is often combined with oculomotor and balance testing.

##### 3.1.2.1 Establishing the presence of dysfunction in the upper cervical spine

###### 3.1.2.1.1 Cervical active movements

Active cervical movements are evaluated to detect cervical spine dysfunctions such as reduced range of motion (ROM) and pain provocation. In the assessment of patients with dizziness, it is crucial to note any dizziness or pain that occurs during tests movements.

Studies have reported reduced cervical ROM in patients with cervicogenic dizziness (88, 116, 117). These measurements were taken prior to treatment, and showed improvements in ROM following intervention. When compared to the normative values of asymptomatic controls (118), the ROM in patients with cervicogenic dizziness appears to be limited. Conversely, Malmstrom et al. (119), observed normal or even increased cervical ROM compared to age-matched normative values, despite the presence of joint tenderness. This discrepancy may result from reduced articular stabilisation, though it is worth noting that the study participants were relatively young (mean age: 37 years). In a comparison of active cervical ROM (ACROM) between dizzy patients, De Vestel et al. found that the ROM did not differ significantly between chronic dizzy patients and those with CGD. However, both groups exhibited reduced cervical extension ROM (40).

While some critique has been raised regarding tests involving head movements due to their stimulation of the vestibular system, it is essential to recognize that these movements inherently engage cervical motion, thereby activating the cervical proprioceptive system. During the terminal phase of movement, proprioceptive impairments may arise, potentially causing patients to experience sensations of dizziness. Thus, the critique might be reframed, suggesting that the debate regarding vestibular versus cervical origins of dizziness may not always benefit patient care but should encourage clinicians to critically interpret their test findings. Also, in patients with obvious vestibular causes for dizziness, a well functioning cervical spine will contribute to optimal vestibular compensation mechanisms.

###### 3.1.2.1.2 Cervical spine palpation and accessory movement testing

Passive functional examination can include palpation of muscles and zygapophyseal joints, for instance using the passive accessory intervertebral movement testing as described by Maitland (120, 121). Local muscle tenderness or tightness of the dorsal neck muscles can indicate cervicogenic dizziness (35, 111, 119, 122). Reproduction of dizziness or pain, or a feeling of restricted joint movement is considered a positive sign and consistent with cervicogenic dizziness (36, 117, 119). Reduced cervical mobility upon palpation of the cervical spine has been reported in suspected cases (16).

In comparison with patients with BPPV, patients with CGD demonstrated more pain on cervical segments and musculature during cervical spine examination (108).

###### 3.1.2.1.3 Joint position error

The joint position relocation test assesses proprioceptive input and motor response. Larger joint position errors or JPEs correspond with higher proprioceptive deficits. When impaired proprioception is the underlying pathophysiological mechanism of cervicogenic dizziness, positive findings on this variable can be expected.

In asymptomatic subjects, increased JPEs have been reported after experimentally inducing pain and after inducing muscular fatigue, indicating that pain and fatigue can alter cervical proprioception (70, 71).

When comparing patients with CGD with healthy controls, Micarelli et al. (88) found higher JPE after left and right rotation and after flexion and extension.

Patients with CGD demonstrate greater JPEs compared to those with BPPV, with reported sensitivity and specificity of 72 and 75%, respectively (108). When applying a threshold of >4,5°, the sensitivity increases to 92%, although specificity decreases to 54%. However, no significant differences in JPE were found between patients with CGD and other chronic dizzy patients or asymptomatic controls (40).

While measuring JPE can be a measure for joint position sense and cervical spine proprioception, these deficits are not unique for cervicogenic dizziness, and may also be present in other conditions such as neck pain, WAD or vestibular disorders. Moreover, patients with vestibular deficits are comparable with controls, suggesting neck movements at low speed are well supplied by proprioceptive inputs (123). More research is therefore needed, with appropriate performance of the JPE test, for instance at different speeds.

##### 3.1.2.2 Testing oculomotor function

Evaluating oculomotor function in patients with suspected cervicogenic dizziness helps to determine if abnormal cervical input leads to abnormalities in gaze stabilising systems like the VOR and COR, which integrate signals from the cervical spine and the vestibular system to stabilise vision during head movements. In the context of cervicogenic dizziness, the head is held still to reduce vestibular input.

###### 3.1.2.2.1 Trunk head co-ordination test

Test subjects turn their body to the right (with head held still), back to the centre, then to the left and back to the centre (holding 30 s in each position). Symptoms related to the cervical spine will be worse in torsion than in neutral, as cervical afferents are isolated. There will be no or less symptoms in “enbloc” rotation (where the whole body rotates).

This test can be used to measure nystagmus in response to cervical neck rotation. Compared to patients with BPPV, those with CGD exhibited nystagmus of more than 2 degrees per second during the cervical rotation torsion test, with a sensitivity of 72% and specificity of 92% (108). Reiley et al. (36) identified the cervical rotation torsion test as having the strongest diagnostic utility to rule in the diagnosis of cervicogenic dizziness.

Treleaven et al., later investigated clinical variants of this test by assessing the occurrence of symptoms during or immediately after the tests. These symptoms included dizziness, visual disturbance, unusual eye movements on opening the eyes after the test, speech disturbance, motion sickness, nausea, slurred speech, dysphagia, light-headedness, tinnitus, headache, or paraesthesia (124). However, they did not compare their findings with other dizzy patients, making it hard to assess its diagnostic value.

###### 3.1.2.2.2 Smooth pursuit neck torsion test

The SPNT test has been proposed as a diagnostic tool for cervical dizziness. In the test, eye movements following a moving target are monitored, first with the head in a neutral, forward-facing position and then with the body rotated beneath the head (106, 107). The presence of nystagmus or saccades is considered indicative of cervical dizziness. However, Wrisley et al. (35) noted that up to 50% of people without cervical dysfunction may test positive, suggesting limited specificity. Also, the manoeuvre is primarily useful when dizziness is provoked by cervical rotation, not extension. Extension has been reported to be the most common movement bringing on cervicogenic dizziness, followed by rotation (125).

##### 3.1.2.3 Balance testing

Balance testing helps to identify postural instability due to altered proprioceptive input from the cervical spine. Balance can be tested using a posturography machine or with more simple tests such as the modified Romberg. Posturography has shown that patients with suspected cervicogenic dizziness exhibit distinctive patterns of altered postural control (32).

More recently, Micarelli et al., studied 93 patients with CGD after excluding vestibular disorders like BPPV, Menières disease, and migrainous vertigo. All patients experienced neck pain triggered by movement for at least 3 months. Objective tests included the Visual Head Impulse Test (VHIT), posturography, and cervical range of motion assessment. Compared to 98 controls, patients showed no differences in VHIT but had reduced neck mobility and altered posturography, suggesting reduced proprioceptive function.

Similarly, De Vestel et al., compared 60 chronic dizzy patients—divided into cervicogenic and non-cervicogenic groups—with 42 asymptomatic controls. They assessed cervical range of motion, joint position error, deep cervical flexor function, and balance. Both patient groups showed impaired muscle function and dynamic balance, though non-cervicogenic patients demonstrated greater deficits in static balance. Visual dependency was similar across patient groups.

It appears that balance assessment can play a role in the assessment of patients with suspected cervicogenic dizziness, in conjunction with other clinical tests.

### 3.2 Conservative treatment of cervicogenic dizziness

For patients with proprioceptive cervicogenic dizziness there is moderate evidence that treatment to the neck reduces symptoms (125, 126). Manual therapy to treat cervical joint dysfunction sometimes combined with for the neck specific movement exercises has been proposed for the treatment of dizziness of cervical origin (31, 117, 119, 125, 127–130). When manual therapy is used, it is believed to be of benefit for this condition because it normalises proprioceptive input by restoring normal movement, or releases trigger points in the cervical muscles and reduces pain (35).

The effectiveness of manual therapy interventions has been studied in several systematic reviews. Their conclusion is that there is level 2 evidence to support the use of cervical manual therapy for cervicogenic dizziness (126, 129, 131).

In the included RCTs, the manual therapy interventions were intended to restore cervical function and reduce cervical pain. They consisted of spinal mobilisation, soft tissue techniques, general home exercises and ergonomic adaptations (31, 119), spinal mobilisations according to Mulligan versus a placebo treatment (116, 117, 125, 132), dry needling with cervical isometric exercises versus exercise alone (133), cervical and thoracic manipulations, mobilisations, massage, range of motion exercises versus sham intervention (134), multimodal treatment consisting of spinal mobilisation, myofascial release techniques, stabilising exercise, TENS application versus this multimodal treatment plus Denneroll traction (135), cervical traction manipulation versus no treatment (136); cervical strength and mobilisation exercises with oculomotor training versus no intervention (137). Vestibular therapy has been shown to have no negative effect on pin in patients with traumatic onset cervicogenic dizziness (138).

Thus, several research groups independently have demonstrated positive effects on dizziness symptoms of treatment that was primarily aimed at restoring cervical dysfunction and pain. Rapid head movements are not involved in this, suggesting that the clinical effects are not due to vestibular adaptation. Moreover, vestibular causes of the dizziness were excluded as far as possible in the included RCTs. Therefore, it seems that for dizzy patients where no vestibular cause can be indicated, and where cervicogenic dizziness is suspected, manual therapy is a therapeutic option.

**Figure 2:**
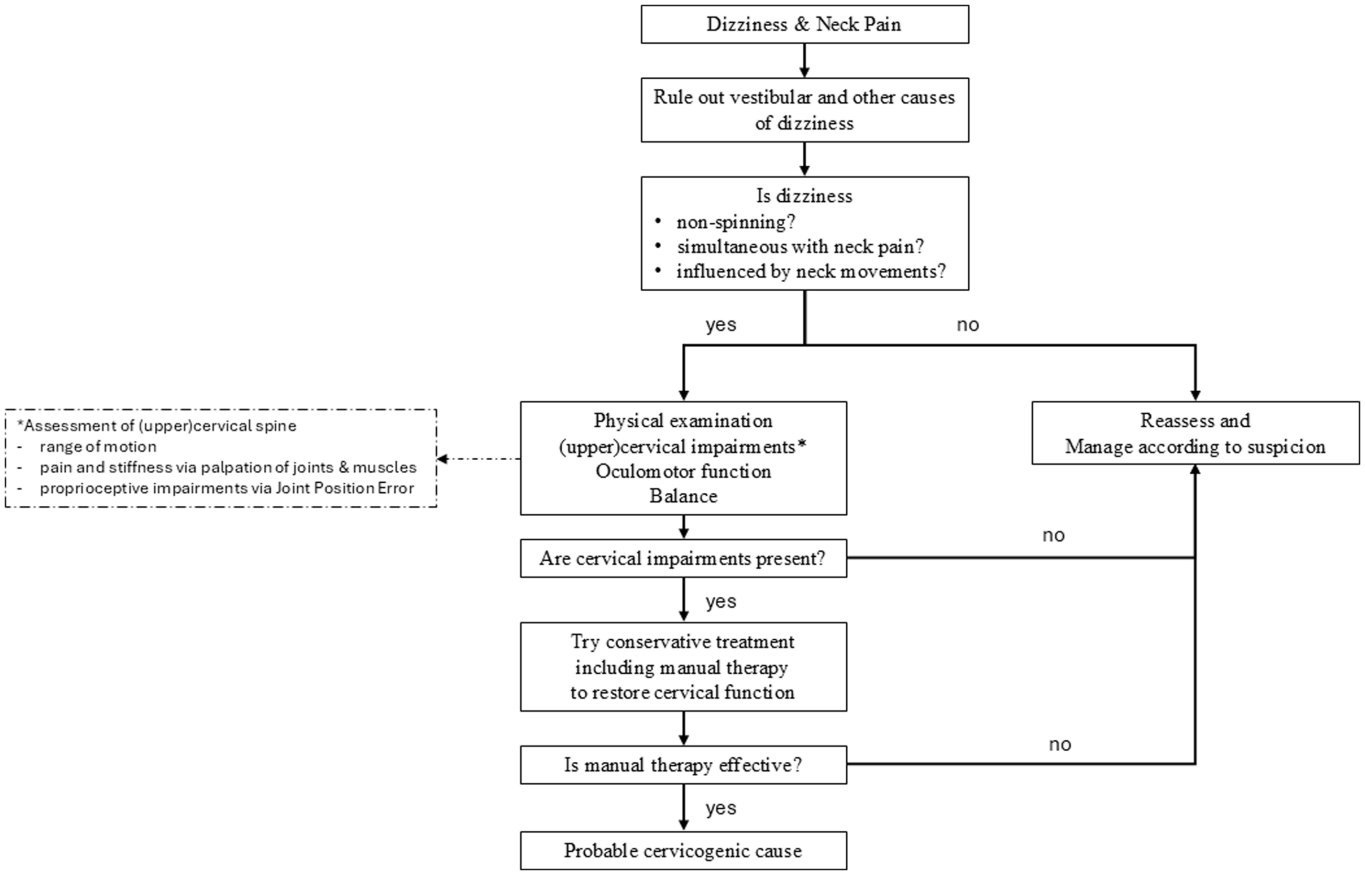
Flowchart to assist clinicians in the assessment and management of patients with suspected cervicogenic dizziness. After excluding other causes of the dizziness, emphasis placed on focused questioning to assess the relationship between dizziness and neck pain. This is followed by a focused physical examination of the (upper) cervical spine and its links to oculomotor functions and balance. If cervical impairments are detected, treatment is directed at addressing these dysfunctions. The treatment outcome helps to confirm or refute a cervicogenic origin of the dizziness.

Figure 2 illustrates a comprehensive flowchart that guides clinicians in diagnosing and managing cervicogenic dizziness, including the evaluation of cervical spine dysfunctions and their relationship with dizziness symptoms.

Additionally, there is evidence that a tailored sensorimotor control program is beneficial in patients with altered cervical related sensorimotor control (head and eye movement control and balance) in reducing symptoms and preventing recurrence (139).

## 4 Discussion/conclusion

Cervicogenic dizziness (CGD) remains a debated diagnosis in both vestibular and musculoskeletal settings. While the coexistence of neck pain and dizziness is well-documented, the recognition of CGD as a distinct clinical entity is inconsistent, partly due to its reliance on exclusionary diagnosis and lack of specific tests.

This paper highlights several key findings regarding cervicogenic dizziness (CGD) and its relationship with cervical proprioception and balance. First, alterations in cervical proprioception, often due to neck pain or dysfunction, can significantly affect postural stability and balance. Cervical pain itself is a major factor in disrupting proprioceptive signals, which are crucial for maintaining sensorimotor control. In some cases, the central nervous system fails to adapt to these altered proprioceptive inputs, leading to inappropriate sensory reweighting and maladaptation. This reweighting can exacerbate symptoms of dizziness, as the brain overly relies on certain sensory inputs at the expense of others.

A critical component of diagnosing CGD is ruling out vestibular causes. The absence of vestibular pathology must be confirmed through comprehensive clinical testing, as many of the signs and symptoms of CGD overlap with vestibular disorders. Therefore, it is essential to interpret all clinical tests within the context of an exclusionary diagnostic approach. By following a stepwise approach – beginning with a detailed history taking and followed by a physical examination of the upper cervical spine, clinicians can more accurately assess the likelihood of CGD.

Therapeutically, reducing cervical pain and restoring neck function have shown clinical benefits for CGD patients. Consequently, clinicians managing patients with CGD are encouraged to consider cervical manual therapy as a treatment option. However, despite the well-documented role of proprioceptive impairment in CGD, there remains a notable lack of studies investigating the effects of specific proprioceptive training in treatment protocols. This gap highlights an important area for future research.

Furthermore, given the complexity of maladaptation and sensory reweighting, it is recommended that treatment for CGD should not focus exclusively on one system. Instead, integrated care should be provided, incorporating vestibular rehabilitation along with targeted interventions for the cervical spine. Addressing both the cervical and vestibular components can help optimize sensorimotor integration and improve patient outcomes. The extent to which each of the systems should receive attention is based on the clinical examination.

Despite advances in understanding CGD, significant gaps remain. Chief among them is the lack of validated diagnostic criteria with sufficient sensitivity and specificity. According to the most recent position paper (21) future research on management strategies should place particular emphasis on carefully defining in-and exclusion criteria. Current diagnostic tests, such as the Smooth Pursuit Neck Torsion Test and joint position error assessments, provide valuable insights but are insufficiently specific to CGD. More research is needed to refine these tests and develop a standardized diagnostic approach.

Additionally, more investigation into central maladaptation is warranted. Understanding how prolonged cervical dysfunction affects sensory processing and central nervous system reorganization may reveal why some individuals with neck pain develop CGD while others do not. Such insights could lead to more targeted interventions that address the root causes of sensory reweighting and maladaptation.

## Data Availability

The original contributions presented in the study are included in the article/supplementary material, further inquiries can be directed to the corresponding author.

## References

[1] Neuhauser HK. The epidemiology of dizziness and vertigo. Handb Clin Neurol. (2016) 137:67–82. doi: 10.1016/B978-0-444-63437-5.00005-4, PMID: 27638063

[2] Bosner S Schwarm S Grevenrath P Schmidt L Horner K Beidatsch D. Prevalence, aetiologies and prognosis of the symptom dizziness in primary care – a systematic review. BMC Fam Pract. (2018) 19:33. doi: 10.1186/s12875-017-0695-0, PMID: 29458336 PMC5819275

[3] Welgampola MS Akdal G Halmagyi GM. Neuro-otology-some recent clinical advances. J Neurol. (2017) 264:188–203. doi: 10.1007/s00415-016-8266-1, PMID: 27632181 PMC5225204

[4] Molnar A McGee S. Diagnosing and treating dizziness. Med Clin North Am. (2014) 98:583–96. doi: 10.1016/j.mcna.2014.01.014, PMID: 24758962

[5] Zhu RT Van Rompaey V Ward BK Van de Berg R Van de Heyning P Sharon JD. The interrelations between different causes of dizziness: a conceptual framework for understanding vestibular disorders. Ann Otol Rhinol Laryngol. (2019) 128:869–78. doi: 10.1177/0003489419845014, PMID: 31018648

[6] Moen U Magnussen LH Wilhelmsen KT Goplen FK Nordahl SHG Meldrum D. Prevalence and distribution of musculoskeletal pain in patients with dizziness-a systematic review. Physiother Res Int. (2022) 27:e1941. doi: 10.1002/pri.1941 35191148 PMC9286866

[7] Malmstrom EM Ekvall Hansson E Hafstrom A Magnusson M Fransson PA. Co-morbidities to vestibular impairments-some concomitant disorders in young and older adults. Front Neurol. (2020) 11:609928. doi: 10.3389/fneur.2020.609928, PMID: 33584509 PMC7873354

[8] Canturk T Bery AK Piccoli D Pyche J Czikk D Osborne J. Longitudinal patient outcomes in chronic dizziness: a scoping review. Otol Neurotol. (2023) 44:848–52. doi: 10.1097/MAO.0000000000004000, PMID: 37703893

[9] Spiegel R Rust H Baumann T Friedrich H Sutter R Goldlin M. Treatment of dizziness: an interdisciplinary update. Swiss Med Wkly. (2017) 147:w14566. doi: 10.4414/smw.2017.14566, PMID: 29282702

[10] Lacour M Helmchen C Vidal PP. Vestibular compensation: the neuro-otologist’s best friend. J Neurol. (2016) 263:54–64. doi: 10.1007/s00415-015-7903-4, PMID: 27083885 PMC4833803

[11] Wuyts FL Van Rompaey V Maes LK. “SO STONED”: common sense approach of the dizzy patient. Front Surg. (2016) 3:32. doi: 10.3389/fsurg.2016.00032, PMID: 27313999 PMC4887462

[12] Micarelli A Viziano A Della-Morte D Augimeri I Alessandrini M. Degree of functional impairment associated with vestibular hypofunction among older adults with cognitive decline. Otol Neurotol. (2018) 39:e392–400. doi: 10.1097/MAO.0000000000001746, PMID: 29547458

[13] Castro P Bancroft MJ Arshad Q Kaski D. Persistent postural-perceptual dizziness (PPPD) from brain imaging to behaviour and perception. Brain Sci. (2022) 12:753. doi: 10.3390/brainsci12060753, PMID: 35741638 PMC9220882

[14] De Vestel C De Hertogh W Van Rompaey V Vereeck L. Comparison of clinical balance and visual dependence tests in patients with chronic dizziness with and without persistent postural-perceptual dizziness: a cross-sectional study. Front Neurol. (2022) 13:880714. doi: 10.3389/fneur.2022.880714, PMID: 35685740 PMC9170888

[15] Staab JP Eckhardt-Henn A Horii A Jacob R Strupp M Brandt T. Diagnostic criteria for persistent postural-perceptual dizziness (PPPD): consensus document of the committee for the classification of vestibular disorders of the Barany society. J Vestib Res. (2017) 27:191–208. doi: 10.3233/VES-170622, PMID: 29036855 PMC9249299

[16] Mb RS Nedison G Garcia CB. Clinical evaluation of neck in patients with proprioceptive Cervicogenic dizziness. Int Tinnitus J. (2022) 25:143–8. doi: 10.5935/0946-5448.20210026, PMID: 35239297

[17] Malmstrom EM Magnusson M Holmberg J Karlberg M Fransson PA. Dizziness and localized pain are often concurrent in patients with balance or psychological disorders. Scand J Pain. (2020) 20:353–62. doi: 10.1515/sjpain-2019-0121, PMID: 31881001

[18] Knapstad MK Ask T Skouen JS Goplen FK Nordahl SHG. Prevalence and consequences of concurrent dizziness on disability and quality of life in patients with long-lasting neck pain. Physiother. Theory Pract. (2022) 39:1266–73. doi: 10.1080/09593985.2022.2034077, PMID: 35152809

[19] De Hertogh W Castien R Jacxsens L De Pauw J Vereeck L. Outcome for dizzy patients in a physiotherapy practice: an observational study. Ann Med. (2022) 54:1787–96. doi: 10.1080/07853890.2022.2091790, PMID: 35786105 PMC9258437

[20] Kazeminasab S Nejadghaderi SA Amiri P Pourfathi H Araj-Khodaei M Sullman MJM. Neck pain: global epidemiology, trends and risk factors. BMC Musculoskelet Disord. (2022) 23:26. doi: 10.1186/s12891-021-04957-4, PMID: 34980079 PMC8725362

[21] Seemungal BM Agrawal Y Bisdorff A Bronstein A Cullen KE Goadsby PJ. The Barany society position on “Cervical Dizziness”. J Vestib Res. (2022) 32:487–99. doi: 10.3233/VES-220202, PMID: 36404562 PMC9837683

[22] Magnusson M Malmstrom EM. The conundrum of cervicogenic dizziness. Handb Clin Neurol. (2016) 137:365–9. doi: 10.1016/B978-0-444-63437-5.00026-1, PMID: 27638084

[23] Hain TC. Cervicogenic causes of vertigo. Curr Opin Neurol. (2015) 28:69–73. doi: 10.1097/WCO.0000000000000161, PMID: 25502050

[24] Chang TP Wang Z Lee XX Kuo YH Schubert MC. Risk of cervical dizziness in patients with cervical spondylosis. JAMA Otolaryngol Head Neck Surg. (2024) 150:93–8. doi: 10.1001/jamaoto.2023.3810, PMID: 38095893 PMC10722389

[25] Martellucci S Attanasio G Ralli M Marcelli V de Vincentiis M Greco A. Does cervical range of motion affect the outcomes of canalith repositioning procedures for posterior canal benign positional paroxysmal vertigo? Am J Otolaryngol. (2019) 40:494–8. doi: 10.1016/j.amjoto.2019.04.003, PMID: 30967256

[26] Micarelli A Viziano A Granito I Arena M Maurizi R Micarelli RX. Onset and resolution failure of recurrent benign paroxysmal positional vertigo: the role of cervical range of motion. Eur Arch Otorrinolaringol. (2022) 279:2183–92. doi: 10.1007/s00405-021-07226-1, PMID: 35091829

[27] Ruhe A Fejer R Walker B. On the relationship between pain intensity and postural sway in patients with non-specific neck pain. J Back Musculoskelet Rehabil. (2013) 26:401–9. doi: 10.3233/BMR-130399, PMID: 23948827

[28] Ruhe A Fejer R Walker B. Altered postural sway in patients suffering from non-specific neck pain and whiplash associated disorder – a systematic review of the literature. Chiropr Man Therap. (2011) 19:13. doi: 10.1186/2045-709X-19-13, PMID: 21609469 PMC3121601

[29] Madsalae T Thongprong T Chinkulprasert C Boonsinsukh R. Can the balance evaluation systems test be used to identify system-specific postural control impairments in older adults with chronic neck pain? Front Med (Lausanne). (2022) 9:1012880. doi: 10.3389/fmed.2022.1012880, PMID: 36388898 PMC9650210

[30] Madsalae T Thongprong T Chaikeeree N Boonsinsukh R. Changes in gait performances during walking with head movements in older adults with chronic neck pain. Front Med (Lausanne). (2024) 11:1324375. doi: 10.3389/fmed.2024.1324375, PMID: 38384408 PMC10879294

[31] Karlberg M Magnusson M Malmstrom EM Melander A Moritz U. Postural and symptomatic improvement after physiotherapy in patients with dizziness of suspected cervical origin. Arch Phys Med Rehabil. (1996) 77:874–82. doi: 10.1016/S0003-9993(96)90273-7, PMID: 8822677

[32] Karlberg M Johansson R Magnusson M Fransson PA. Dizziness of suspected cervical origin distinguished by posturographic assessment of human postural dynamics. J Vestib Res. (1996) 6:37–47. doi: 10.3233/VES-1996-6105, PMID: 8719508

[33] Roy JE Cullen KE. Dissociating self-generated from passively applied head motion: neural mechanisms in the vestibular nuclei. J Neurosci. (2004) 24:2102–11. doi: 10.1523/JNEUROSCI.3988-03.2004, PMID: 14999061 PMC6730417

[34] Mergner T Hlavacka F Schweigart G. Interaction of vestibular and proprioceptive inputs. J Vestib Res. (1993) 3:41–57. PMID: 8275243

[35] Wrisley DM Sparto PJ Whitney SL Furman JM. Cervicogenic dizziness: a review of diagnosis and treatment. J Orthop Sports Phys Ther. (2000) 30:755–66. doi: 10.2519/jospt.2000.30.12.755, PMID: 11153554

[36] Reiley AS Vickory FM Funderburg SE Cesario RA Clendaniel RA. How to diagnose cervicogenic dizziness. Arch Physiother. (2017) 7:12. doi: 10.1186/s40945-017-0040-x, PMID: 29340206 PMC5759906

[37] Luscher M Theilgaard S Edholm B. Prevalence and characteristics of diagnostic groups amongst 1034 patients seen in ENT practices for dizziness. J Laryngol Otol. (2014) 128:128–33. doi: 10.1017/S0022215114000188, PMID: 24521903

[38] Polaczkiewicz L Olszewski J. Analyze causes and results of VNG examinations in patients with vertigo and balance disorders in the private ENT practice. Otolaryngol Pol. (2019) 74:23–30. doi: 10.5604/01.3001.0013.4374, PMID: 32022699

[39] Vural M Karan A Albayrak Gezer I Caliskan A Atar S Yildiz Aydin F. Prevalence, etiology, and biopsychosocial risk factors of cervicogenic dizziness in patients with neck pain: a multi-center, cross-sectional study. Turk J Phys Med Rehabil. (2021) 67:399–408. doi: 10.5606/tftrd.2021.7983, PMID: 35141479 PMC8790272

[40] De Vestel C Vereeck L Van Rompaey V Reid SA De Hertogh W. Clinical characteristics and diagnostic aspects of cervicogenic dizziness in patients with chronic dizziness: a cross-sectional study. Musculoskelet Sci Pract. (2022) 60:102559. doi: 10.1016/j.msksp.2022.102559, PMID: 35364427

[41] Yacovino DA Hain TC. Clinical characteristics of cervicogenic-related dizziness and vertigo. Semin Neurol. (2013) 33:244–55. doi: 10.1055/s-0033-1354592, PMID: 24057828

[42] Li Y Peng B. Pathogenesis, diagnosis, and treatment of cervical Vertigo. Pain Physician. (2015) 18:E583–95. doi: 10.36076/ppj.2015/18/E583, PMID: 26218949

[43] Kristjansson E Treleaven J. Sensorimotor function and dizziness in neck pain: implications for assessment and management. J Orthop Sports Phys Ther. (2009) 39:364–77. doi: 10.2519/jospt.2009.2834, PMID: 19411769

[44] Treleaven J. Sensorimotor disturbances in neck disorders affecting postural stability, head and eye movement control. Man Ther. (2008) 13:2–11. doi: 10.1016/j.math.2007.06.003, PMID: 17702636

[45] Bernard C. Introduction a l’étude de la Médecine Expérimentale. Paris: Baillière et Fils (1856).

[46] Sadeghi SG Minor LB Cullen KE. Neural correlates of sensory substitution in vestibular pathways following complete vestibular loss. J Neurosci. (2012) 32:14685–95. doi: 10.1523/JNEUROSCI.2493-12.2012, PMID: 23077054 PMC3503523

[47] Brooks JX Cullen KE. The primate cerebellum selectively encodes unexpected self-motion. Curr Biol. (2013) 23:947–55. doi: 10.1016/j.cub.2013.04.029, PMID: 23684973 PMC6100740

[48] de Jong PT de Jong JM Cohen B Jongkees LB. Ataxia and nystagmus induced by injection of local anesthetics in the neck. Ann Neurol. (1977) 1:240–6. doi: 10.1002/ana.410010307, PMID: 407834

[49] Biemond A De Jong JM. On cervical nystagmus and related disorders. Brain. (1969) 92:437–58. doi: 10.1093/brain/92.2.437, PMID: 5305995

[50] Ishikawa K Matsuzaki Z Yokomizo M Terada N Miyazaki S Togawa K. Effect of unilateral section of cervical afferent nerve upon optokinetic response and vestibular nystagmus induced by sinusoidal rotation in guinea pigs. Acta Otolaryngol Suppl. (1998) 537:6–10. PMID: 9870641 10.1080/00016489850182279

[51] Lennerstrand G Han Y Velay JL. Properties of eye movements induced by activation of neck muscle proprioceptors. Graefes Arch Clin Exp Ophthalmol. (1996) 234:703–9. doi: 10.1007/BF00292357, PMID: 8950591

[52] Taylor JL McCloskey DI. Illusions of head and visual target displacement induced by vibration of neck muscles. Brain. (1991) 114:755–9. doi: 10.1093/brain/114.2.755, PMID: 2043947

[53] Chalimourdas A Gilles A De Hertogh W Michiels S. Does vibration frequency and location influence the effect of neck muscle vibration on postural sway? A cross-sectional study in asymptomatic participants. Exp Brain Res. (2023) 241:2261–73. doi: 10.1007/s00221-023-06680-z, PMID: 37552270

[54] Kavounoudias A Gilhodes JC Roll R Roll JP. From balance regulation to body orientation: two goals for muscle proprioceptive information processing? Exp Brain Res. (1999) 124:80–8. doi: 10.1007/s002210050602, PMID: 9928792

[55] Xie H Song H Schmidt C Chang WP Chien JH. The effect of mechanical vibration-based stimulation on dynamic balance control and gait characteristics in healthy young and older adults: a systematic review of cross-sectional study. Gait Posture. (2023) 102:18–38. doi: 10.1016/j.gaitpost.2023.02.013, PMID: 36871475

[56] Malmstrom EM Fransson PA Jaxmar Bruinen T Facic S Tjernstrom F. Disturbed cervical proprioception affects perception of spatial orientation while in motion. Exp Brain Res. (2017) 235:2755–66. doi: 10.1007/s00221-017-4993-5, PMID: 28623390 PMC5550524

[57] Kulkarni V Chandy MJ Babu KS. Quantitative study of muscle spindles in suboccipital muscles of human foetuses. Neurol India. (2001) 49:355–9. 11799407

[58] McLain RF. Mechanoreceptor endings in human cervical facet joints. Spine (Phila Pa 1976). (1994) 19:495–501. doi: 10.1097/00007632-199403000-00001, PMID: 8184340

[59] Liu JX Thornell LE Pedrosa-Domellof F. Muscle spindles in the deep muscles of the human neck: a morphological and immunocytochemical study. J Histochem Cytochem. (2003) 51:175–86. doi: 10.1177/002215540305100206, PMID: 12533526

[60] Boyd-Clark LC Briggs CA Galea MP. Muscle spindle distribution, morphology, and density in longus colli and multifidus muscles of the cervical spine. Spine (Phila Pa 1976). (2002) 27:694–701. doi: 10.1097/00007632-200204010-00005, PMID: 11923661

[61] Cullen KE Roy JE. Signal processing in the vestibular system during active versus passive head movements. J Neurophysiol. (2004) 91:1919–33. doi: 10.1152/jn.00988.2003, PMID: 15069088

[62] Falla D. Unravelling the complexity of muscle impairment in chronic neck pain. Man Ther. (2004) 9:125–33. doi: 10.1016/j.math.2004.05.003, PMID: 15245706

[63] Zhou W Tang BF Newlands SD King WM. Responses of monkey vestibular-only neurons to translation and angular rotation. J Neurophysiol. (2006) 96:2915–30. doi: 10.1152/jn.00013.2006, PMID: 16943321

[64] Crane BT. The influence of head and body tilt on human fore-aft translation perception. Exp Brain Res. (2014) 232:3897–905. doi: 10.1007/s00221-014-4060-4, PMID: 25160866 PMC5091300

[65] Wyke B. Cervical articular contributions to posture and gait-their relation to senile disequilibrium. Age Ageing. (1979) 8:251–8. doi: 10.1093/ageing/8.4.251 517320

[66] Mergner T Nardi GL Becker W Deecke L. The role of canal-neck interaction for the perception of horizontal trunk and head rotation. Exp Brain Res. (1983) 49:198–208. doi: 10.1007/BF00238580, PMID: 6832257

[67] Micarelli A Viziano A Augimeri I Micarelli B Capoccia D Alessandrini M. Diagnostic route of cervicogenic dizziness: usefulness of posturography, objective and subjective testing implementation and their correlation. Disabil Rehabil. (2021) 43:1730–7. doi: 10.1080/09638288.2019.1680747, PMID: 31656108

[68] Malmstrom EM Stjerna J Hogestatt ED Westergren H. Quantitative sensory testing of temperature thresholds: possible biomarkers for persistent pain? J Rehabil Med. (2016) 48:43–7. doi: 10.2340/16501977-2024, PMID: 26450179

[69] Michiels S De Hertogh W Truijen S November D Wuyts F Van de Heyning P. The assessment of cervical sensory motor control: a systematic review focusing on measuring methods and their clinimetric characteristics. Gait Posture. (2013) 38:1–7. doi: 10.1016/j.gaitpost.2012.10.007, PMID: 23153836

[70] Malmstrom EM Karlberg M Holmstrom E Fransson PA Hansson GA Magnusson M. Influence of prolonged unilateral cervical muscle contraction on head repositioning – decreased overshoot after a 5-min static muscle contraction task. Man Ther. (2010) 15:229–34. doi: 10.1016/j.math.2009.12.003, PMID: 20083423

[71] Malmstrom EM Westergren H Fransson PA Karlberg M Magnusson M. Experimentally induced deep cervical muscle pain distorts head on trunk orientation. Eur J Appl Physiol. (2013) 113:2487–99. doi: 10.1007/s00421-013-2683-y, PMID: 23812089

[72] Feipel V Salvia P Klein H Rooze M. Head repositioning accuracy in patients with whiplash-associated disorders. Spine (Phila Pa 1976). (2006) 31:E51–8. doi: 10.1097/01.brs.0000194786.63690.54, PMID: 16418625

[73] Revel M Andre-Deshays C Minguet M. Cervicocephalic kinesthetic sensibility in patients with cervical pain. Arch Phys Med Rehabil. (1991) 72:288–91. PMID: 2009044

[74] Loudon JK Ruhl M Field E. Ability to reproduce head position after whiplash injury. Spine (Phila Pa 1976). (1997) 22:865–8. doi: 10.1097/00007632-199704150-00008, PMID: 9127919

[75] Sterling M Jull G Vicenzino B Kenardy J Darnell R. Development of motor system dysfunction following whiplash injury. Pain. (2003) 103:65–73. doi: 10.1016/S0304-3959(02)00420-7, PMID: 12749960

[76] Lee HY Wang JD Yao G Wang SF. Association between cervicocephalic kinesthetic sensibility and frequency of subclinical neck pain. Man Ther. (2008) 13:419–25. doi: 10.1016/j.math.2007.04.001, PMID: 17544825

[77] Brandt T Bronstein AM. Cervical vertigo. J Neurol Neurosurg Psychiatry. (2001) 71:8–12. doi: 10.1136/jnnp.71.1.8, PMID: 11413255 PMC1737478

[78] Passatore M Roatta S. Influence of sympathetic nervous system on sensorimotor function: whiplash associated disorders (WAD) as a model. Eur J Appl Physiol. (2006) 98:423–49. doi: 10.1007/s00421-006-0312-8, PMID: 17036216

[79] Johansson H Sojka P. Pathophysiological mechanisms involved in genesis and spread of muscular tension in occupational muscle pain and in chronic musculoskeletal pain syndromes: a hypothesis. Med Hypotheses. (1991) 35:196–203. doi: 10.1016/0306-9877(91)90233-O, PMID: 1943863

[80] Sjolander P Johansson H Djupsjobacka M. Spinal and supraspinal effects of activity in ligament afferents. J Electromyogr Kinesiol. (2002) 12:167–76. doi: 10.1016/S1050-6411(02)00017-2, PMID: 12086810

[81] Yang L Chen J Yang C Pang X Li D Wu B. Cervical intervertebral disc degeneration contributes to dizziness: a clinical and Immunohistochemical study. World Neurosurg. (2018) 119:e686–93. doi: 10.1016/j.wneu.2018.07.243, PMID: 30092465

[82] Yang L Yang C Pang X Li D Yang H Zhang X. Mechanoreceptors in diseased cervical intervertebral disc and Vertigo. Spine (Phila Pa 1976). (2017) 42:540–6. doi: 10.1097/BRS.0000000000001801, PMID: 27438387

[83] Reddy RS Tedla JS Dixit S Abohashrh M. Cervical proprioception and its relationship with neck pain intensity in subjects with cervical spondylosis. BMC Musculoskelet Disord. (2019) 20:447. doi: 10.1186/s12891-019-2846-z, PMID: 31615495 PMC6794723

[84] Elliott J Jull G Noteboom JT Darnell R Galloway G Gibbon WW. Fatty infiltration in the cervical extensor muscles in persistent whiplash-associated disorders: a magnetic resonance imaging analysis. Spine (Phila Pa 1976). (2006) 31:E847–55. doi: 10.1097/01.brs.0000240841.07050.34, PMID: 17047533

[85] Gill-Lussier J Saliba I Barthelemy D. Proprioceptive Cervicogenic dizziness care trajectories in patient subpopulations: a scoping review. J Clin Med. (2023) 12:1884. doi: 10.3390/jcm12051884, PMID: 36902670 PMC10003866

[86] Li Y Yang L Dai C Peng B. Proprioceptive Cervicogenic dizziness: a narrative review of pathogenesis, diagnosis, and treatment. J Clin Med. (2022) 11:6293. doi: 10.3390/jcm11216293, PMID: 36362521 PMC9655761

[87] Wu B Yang L Peng B. Ingrowth of nociceptive receptors into diseased cervical intervertebral disc is associated with Discogenic neck pain. Pain Med. (2019) 20:1072–7. doi: 10.1093/pm/pnz013, PMID: 30848823

[88] Micarelli A Viziano A Carlino P Granito I Micarelli RX Alessandrini M. Reciprocal roles of joint position error, visual dependency and subjective perception in cervicogenic dizziness. Somatosens Mot Res. (2020) 37:262–70. doi: 10.1080/08990220.2020.1803257, PMID: 32772608

[89] Riemann BL Lephart SM. The sensorimotor system, part I: the physiologic basis of functional joint stability. J Athl Train. (2002) 37:71–9. PMID: 16558670 PMC164311

[90] Gdowski GT McCrea RA. Neck proprioceptive inputs to primate vestibular nucleus neurons. Exp Brain Res. (2000) 135:511–26. doi: 10.1007/s002210000542, PMID: 11156315

[91] Armstrong B McNair P Taylor D. Head and neck position sense. Sports Med. (2008) 38:101–17. doi: 10.2165/00007256-200838020-00002, PMID: 18201114

[92] Matsushita M Gao X Yaginuma H. Spinovestibular projections in the rat, with particular reference to projections from the central cervical nucleus to the lateral vestibular nucleus. J Comp Neurol. (1995) 361:334–44. doi: 10.1002/cne.903610210, PMID: 8543666

[93] Dutia MB. The muscles and joints of the neck: their specialisation and role in head movement. Prog Neurobiol. (1991) 37:165–78. doi: 10.1016/0301-0082(91)90026-W, PMID: 1947176

[94] Hongo T Kitama T Yoshida K. Integration of vestibular and neck afferent signals in the central cervical nucleus. Prog Brain Res. (1988) 76:155–62. doi: 10.1016/S0079-6123(08)64501-X, PMID: 3064141

[95] Pompeiano O. Spinovestibular relations: anatomical and physiological aspects. Prog Brain Res. (1972) 37:263–96. doi: 10.1016/S0079-6123(08)63907-2, PMID: 4345124

[96] McCall AA Miller DM Yates BJ. Descending influences on Vestibulospinal and Vestibulosympathetic reflexes. Front Neurol. (2017) 8:112. doi: 10.3389/fneur.2017.00112, PMID: 28396651 PMC5366978

[97] Balaban CD. Vestibular nucleus projections to the parabrachial nucleus in rabbits: implications for vestibular influences on the autonomic nervous system. Exp Brain Res. (1996) 108:367–81. doi: 10.1007/BF00227260, PMID: 8801117

[98] Radovanovic D Peikert K Lindstrom M Domellof FP. Sympathetic innervation of human muscle spindles. J Anat. (2015) 226:542–8. doi: 10.1111/joa.12309, PMID: 25994126 PMC4450958

[99] Richmond FJ Abrahams VC. Physiological properties of muscle spindles in dorsal neck muscles of the cat. J Neurophysiol. (1979) 42:604–17. doi: 10.1152/jn.1979.42.2.604, PMID: 154558

[100] Humphreys BK. Cervical outcome measures: testing for postural stability and balance. J Manip Physiol Ther. (2008) 31:540–6. doi: 10.1016/j.jmpt.2008.08.007, PMID: 18804005

[101] Angelaki DE Cullen KE. Vestibular system: the many facets of a multimodal sense. Annu Rev Neurosci. (2008) 31:125–50. doi: 10.1146/annurev.neuro.31.060407.125555, PMID: 18338968

[102] Tinazzi M Fiaschi A Rosso T Faccioli F Grosslercher J Aglioti SM. Neuroplastic changes related to pain occur at multiple levels of the human somatosensory system: a somatosensory-evoked potentials study in patients with cervical radicular pain. J Neurosci. (2000) 20:9277–83. doi: 10.1523/JNEUROSCI.20-24-09277.2000, PMID: 11125006 PMC6773009

[103] Gosselin G Fagan MJ. Effects of cervical muscle fatigue on the perception of the subjective vertical and horizontal. Springerplus. (2014) 3:78. doi: 10.1186/2193-1801-3-78, PMID: 24600540 PMC3940717

[104] Brady RA Peters BT Batson CD Ploutz-Snyder R Mulavara AP Bloomberg JJ. Gait adaptability training is affected by visual dependency. Exp Brain Res. (2012) 220:1–9. doi: 10.1007/s00221-012-3109-5, PMID: 22585123

[105] Grod JP Diakow PR. Effect of neck pain on verticality perception: a cohort study. Arch Phys Med Rehabil. (2002) 83:412–5. doi: 10.1053/apmr.2002.29660, PMID: 11887124

[106] Tjell C Rosenhall U. Smooth pursuit neck torsion test: a specific test for cervical dizziness. Am J Otol. (1998) 19:76–81. PMID: 9455954

[107] Treleaven J Jull G LowChoy N. Smooth pursuit neck torsion test in whiplash-associated disorders: relationship to self-reports of neck pain and disability, dizziness and anxiety. J Rehabil Med. (2005) 37:219–23. doi: 10.1080/16501970410024299, PMID: 16024477

[108] L’Heureux-Lebeau B Godbout A Berbiche D Saliba I. Evaluation of paraclinical tests in the diagnosis of cervicogenic dizziness. Otol Neurotol. (2014) 35:1858–65. doi: 10.1097/MAO.0000000000000506, PMID: 25058834

[109] Brandt T Strupp M Dieterich M. Five keys for diagnosing most vertigo, dizziness, and imbalance syndromes: an expert opinion. J Neurol. (2014) 261:229–31. doi: 10.1007/s00415-013-7190-x, PMID: 24292642

[110] Roland LT Kallogjeri D Sinks BC Rauch SD Shepard NT White JA. Utility of an abbreviated dizziness questionnaire to differentiate between causes of Vertigo and guide appropriate referral: a multicenter prospective blinded study. Otol Neurotol. (2015) 36:1687–94. doi: 10.1097/MAO.0000000000000884, PMID: 26485598 PMC4692465

[111] Kondratek M Creighton D Krauss J. Use of Translatoric mobilization in a patient with Cervicogenic dizziness and motion restriction: a case report. J Man Manip Ther. (2006) 14:140–51. doi: 10.1179/106698106790835696

[112] Kerber KA Newman-Toker DE. Misdiagnosing dizzy patients: common pitfalls in clinical practice. Neurol Clin. (2015) 33:565–575, viii. doi: 10.1016/j.ncl.2015.04.009, PMID: 26231272 PMC9023124

[113] Nacci A Ferrazzi M Berrettini S Panicucci E Matteucci J Bruschini L. Vestibular and stabilometric findings in whiplash injury and minor head trauma. Acta Otorhinolaryngol Ital. (2011) 31:378–89. PMID: 22323849 PMC3272873

[114] Treleaven J Peterson G Ludvigsson ML Peolsson A. Cervical musculoskeletal, physical and psychological factors associated with ongoing dizziness in patients with whiplash associated disorder, 12 months after undertaking a neck specific or general exercise intervention. BMC Musculoskelet Disord. (2022) 23:683. doi: 10.1186/s12891-022-05642-w, PMID: 35850745 PMC9290277

[115] Reneker JC Cheruvu VK Yang J James MA Cook CE. Physical examination of dizziness in athletes after a concussion: a descriptive study. Musculoskelet Sci Pract. (2018) 34:8–13. doi: 10.1016/j.msksp.2017.11.012, PMID: 29197811

[116] Reid SA Callister R Katekar MG Rivett DA. Effects of cervical spine manual therapy on range of motion, head repositioning, and balance in participants with cervicogenic dizziness: a randomized controlled trial. Arch Phys Med Rehabil. (2014) 95:1603–12. doi: 10.1016/j.apmr.2014.04.009, PMID: 24792139

[117] Reid SA Rivett DA Katekar MG Callister R. Sustained natural apophyseal glides (SNAGs) are an effective treatment for cervicogenic dizziness. Man Ther. (2008) 13:357–66. doi: 10.1016/j.math.2007.03.006, PMID: 17951095

[118] Swinkels RA Swinkels-Meewisse IE. Normal values for cervical range of motion. Spine (Phila Pa 1976). (2014) 39:362–7. doi: 10.1097/BRS.0000000000000158, PMID: 24573069

[119] Malmstrom EM Karlberg M Melander A Magnusson M Moritz U. Cervicogenic dizziness – musculoskeletal findings before and after treatment and long-term outcome. Disabil Rehabil. (2007) 29:1193–205. doi: 10.1080/09638280600948383, PMID: 17653993

[120] Maitland G. Vertebral manipulation. 4th Edition ed. London: Butterworth Heineman (1977).

[121] Maitland GD. Vertebral manipulation. 8th ed. Amsterdam: Elsevier (2013).

[122] Hinoki M. Vertigo due to whiplash injury: a neurotological approach. Acta Otolaryngol. (1985) 98:9–29. doi: 10.1080/00016489.1985.12005652, PMID: 6599233

[123] Malmstrom EM Karlberg M Fransson PA Lindbladh J Magnusson M. Cervical proprioception is sufficient for head orientation after bilateral vestibular loss. Eur J Appl Physiol. (2009) 107:73–81. doi: 10.1007/s00421-009-1097-3, PMID: 19506897

[124] Treleaven J Joloud V Nevo Y Radcliffe C Ryder M. Normative responses to clinical tests for Cervicogenic dizziness: clinical cervical torsion test and head-neck differentiation test. Phys Ther. (2020) 100:192–200. doi: 10.1093/ptj/pzz143, PMID: 31584656

[125] Reid SA Callister R Snodgrass SJ Katekar MG Rivett DA. Manual therapy for cervicogenic dizziness: long-term outcomes of a randomised trial. Man Ther. (2015) 20:148–56. doi: 10.1016/j.math.2014.08.003, PMID: 25220110

[126] De Vestel C Vereeck L Reid SA Van Rompaey V Lemmens J De Hertogh W. Systematic review and meta-analysis of the therapeutic management of patients with cervicogenic dizziness. J Man Manip Ther. (2022) 30:273–83. doi: 10.1080/10669817.2022.2033044, PMID: 35383538 PMC9487935

[127] Galm R Rittmeister M Schmitt E. Vertigo in patients with cervical spine dysfunction. Eur Spine J. (1998) 7:55–8. doi: 10.1007/s005860050028, PMID: 9548360 PMC3615355

[128] Heikkila H Johansson M Wenngren BI. Effects of acupuncture, cervical manipulation and NSAID therapy on dizziness and impaired head repositioning of suspected cervical origin: a pilot study. Man Ther. (2000) 5:151–7. doi: 10.1054/math.2000.0357, PMID: 11034885

[129] Lystad RP Bell G Bonnevie-Svendsen M Carter CV. Manual therapy with and without vestibular rehabilitation for cervicogenic dizziness: a systematic review. Chiropr Man Therap. (2011) 19:21. doi: 10.1186/2045-709X-19-21, PMID: 21923933 PMC3182131

[130] Reid SA Rivett DA. Manual therapy treatment of cervicogenic dizziness: a systematic review. Man Ther. (2005) 10:4–13. doi: 10.1016/j.math.2004.03.006, PMID: 15681263

[131] Yaseen K Hendrick P Ismail A Felemban M Alshehri MA. The effectiveness of manual therapy in treating cervicogenic dizziness: a systematic review. J Phys Ther Sci. (2018) 30:96–102. doi: 10.1589/jpts.30.96, PMID: 29410575 PMC5788784

[132] Micarelli A Viziano A Granito I Carlino P Micarelli RX Augimeri I. Postural and clinical outcomes of sustained natural apophyseal glides treatment in cervicogenic dizziness patients: a randomised controlled trial. Clin Rehabil. (2021) 35:1566–76. doi: 10.1177/02692155211012413, PMID: 33896213

[133] Aydin T Dernek B Senturk Ege T Karan A Aksoy C. The effectiveness of dry needling and exercise therapy in patients with dizziness caused by cervical myofascial pain syndrome; Prospective randomized clinical study. Pain Med. (2019) 20:153–60. doi: 10.1093/pm/pny072, PMID: 29718418

[134] Kendall JC French SD Hartvigsen J Azari MF. Chiropractic treatment including instrument-assisted manipulation for non-specific dizziness and neck pain in community-dwelling older people: a feasibility randomised sham-controlled trial. Chiropr Man Therap. (2018) 26:14. doi: 10.1186/s12998-018-0183-1 PMC5943997 29760878

[135] Moustafa IM Diab AA Harrison DE. The effect of normalizing the sagittal cervical configuration on dizziness, neck pain, and cervicocephalic kinesthetic sensibility: a 1-year randomized controlled study. Eur J Phys Rehabil Med. (2017) 53:57–71. doi: 10.23736/S1973-9087.16.04179-4, PMID: 27575013

[136] Carrasco-Uribarren A Rodriguez-Sanz J Lopez-de-Celis C Fanlo-Mazas P Cabanillas-Barea S. An upper cervical spine treatment protocol for cervicogenic dizziness: a randomized controlled trial. Physiother Theory Pract. (2022) 38:2640–9. doi: 10.1080/09593985.2021.1972500, PMID: 34496721

[137] Piromchai P Toumjaidee N Srirompotong S Yimtae K. The efficacy of self-exercise in a patient with cervicogenic dizziness: a randomized controlled trial. Front Neurol. (2023) 14:1121101. doi: 10.3389/fneur.2023.1121101, PMID: 36864911 PMC9972221

[138] Hansson EE Persson L Malmstrom EM. Influence of vestibular rehabilitation on neck pain and cervical range of motion among patients with whiplash-associated disorder: a randomized controlled trial. J Rehabil Med. (2013) 45:906–10. doi: 10.2340/16501977-1197, PMID: 23974698

[139] Sremakaew M Jull G Treleaven J Uthaikhup S. Effectiveness of adding rehabilitation of cervical related sensorimotor control to manual therapy and exercise for neck pain: a randomized controlled trial. Musculoskelet Sci Pract. (2023) 63:102690. doi: 10.1016/j.msksp.2022.102690, PMID: 36414518

